# Catheter-Based Therapies in Acute Pulmonary Embolism—Mortality and Safety Outcomes: A Systematic Review and Meta-Analysis

**DOI:** 10.3390/jcm14124167

**Published:** 2025-06-12

**Authors:** Panagiotis Zoumpourlis, Shaunak Mangeshkar, Kuan-Yu Chi, Dimitrios Varrias, Michail Spanos, Muhammad Fahimuddin, Matthew D. Langston, Usman A. Khan, Michael J. Grushko, Prabhjot Singh, Seth I. Sokol

**Affiliations:** NYC Health + Hospitals, Jacobi Hospital, Albert Einstein College of Medicine, Bronx, NY 10461, USA; mangeshs@nychhc.org (S.M.); chik1@nychhc.org (K.-Y.C.); varriasd@nychhc.org (D.V.); spanosm@nychhc.org (M.S.); fahimudm@nychhc.org (M.F.); matthew.langston@nychhc.org (M.D.L.); khanu7@nychhc.org (U.A.K.); michael.grushko@nychhc.org (M.J.G.); singhp8@nychhc.org (P.S.); seth.sokol@nychhc.org (S.I.S.)

**Keywords:** catheter-based therapy, acute pulmonary embolism, catheter-directed thrombolysis, mechanical thrombectomy, intermediate-risk pulmonary embolism

## Abstract

**Background/Objectives:** Right ventricular (RV) dysfunction and circulatory collapse are considered to play a major role in mortality in patients suffering from pulmonary embolism (PE). Catheter-based therapies (CBTs) have been shown to improve RV hemodynamics. The aim of this study was to present available data for CBTs in acute PE and investigate whether CBTs offer mortality benefit and better safety outcomes over anticoagulation (AC) in patients with intermediate-risk PE. **Methods:** PubMed was searched from inception until February 2024 for studies that investigated treatment strategies in patients with confirmed PE. We initially investigated the crude incidence of mortality and major bleeding for individual interventions in patients with either intermediate or high-risk PE. We then directly compared CBT to AC for intermediate-risk PE, for which the effectiveness endpoint was comparative short-term (30-day or in-hospital) and midterm (90-day or 1-year) all-cause mortality and the safety outcomes included minor bleeding, major bleeding, and intracranial hemorrhage (ICH). **Results:** In all, 59 studies (4457 patients) were eventually included in our study. For the prevalence study, we described the crude incidence for mortality and major bleeding for interventions like catheter-directed thrombolysis (CDT), mechanical thrombectomy (MT), AC, and systemic thrombolysis (ST) in patients with either intermediate or high-risk PE. Our data synthesis comparing CBT to AC included 1657 patients (11 studies) with intermediate-risk PE. Our results indicate that CBT is associated with a lower risk of both short-term (RR 0.43; 95% CI [0.24–0.79], I^2^ = 0%) and midterm all-cause mortality (RR 0.38; 95% CI [0.23–0.62], I^2^ = 0%) compared to AC. Major bleeding and ICH did not differ between the two groups. **Conclusions:** In patients with intermediate-risk PE, our meta-analysis of the current literature suggests that CBT offers better outcomes in terms of short-term and midterm mortality compared to AC alone, with no difference in safety outcomes. Further RCTs are needed to explore and validate these findings.

## 1. Introduction

Pulmonary embolism (PE) is a life-threatening manifestation of venous thromboembolism (VTE) that can be challenging to diagnose and treat. Patients with PE can have variable presentations ranging from cardiopulmonary arrest to minimally symptomatic or asymptomatic. PE severity can be classified as high-risk or massive, intermediate-risk or submassive, and low-risk according to the American Heart Association (AHA) and the European Society of Cardiology (ESC) [1,2]. High-risk PE refers to patients presenting with hypotension, defined as systolic blood pressure < 90 mmHg, a drop of >40 mmHg for 15 minutes, or the need for vasopressor support. Current guidelines favor the use of systemic thrombolysis (ST) over catheter-based therapies (CBT) in patients with high-risk PE, recommending CBT when ST has failed or is contraindicated [1,3,4,5,6,7]. Intermediate-risk PE refers to patients with right ventricular (RV) strain with no hypotension according to ESC. Similarly, AHA defines submassive PE as acute PE without systemic hypotension (systolic blood pressure >90 mmHg) but with either RV dysfunction or myocardial necrosis. In these patients, guidelines recommend initial anticoagulation (AC) and may consider ST if there is hemodynamic deterioration, leaving CBT as an alternative [3,8].

Intermediate-risk PE can be further subclassified based on the degree of RV dysfunction and the presence of cardiac biomarkers [3]. Intermediate-high risk patients have evidence of RV dysfunction on initial echocardiography or CT pulmonary angiography, which puts them at risk for increased 30-day mortality, which can reach up to 10% [3]. If there is no RV dysfunction on imaging and or no elevation in cardiac biomarkers, PE is classified as intermediate–low risk, and 30-day mortality can be as low as 1% [3]. Considering the above, it appears that RV dysfunction and circulatory collapse play a major role in mortality in patients with PE [9], and therapies targeted at reducing RV afterload could potentially reduce mortality or prevent progression to hemodynamic deterioration. It has been demonstrated that RV/LV ratio >0.9 is independently associated with increased 30-day mortality [10] and certain CBTs have been shown to improve outcomes such as the RV/LV ratio and systolic pulmonary artery pressure (sPAP) which are used to assess RV afterload and dysfunction [11,12].

In this study, we first describe outcomes of the available interventions for acute PE, including anticoagulation, systemic thrombolysis, and catheter-based therapies (CBTs). We then compare the outcomes of CBT and AC in patients with intermediate-risk pulmonary embolism in an effort to investigate the above hypothesis of whether acute interventions aiming at improving RV function can improve mortality. 

## 2. Materials and Methods

We conducted this systematic review and meta-analysis in accordance with the Cochrane Handbook for Systematic Reviews of Interventions. We reported the study results in accordance with the Preferred Reporting Items for Systematic Reviews and Meta-Analyses (PRISMA). The review protocol was not registered.

### 2.1. Study Selection and Eligibility Criteria

We systematically reviewed the literature in PubMed from inception until February 2024 to identify studies that investigated treatment strategies in patients with confirmed PE of at least intermediate risk who underwent any form of CBT including catheter-directed thrombolysis (CDT) or mechanical thrombectomy (MT). The search utilized the following terms: “Thrombectomy” AND “Thrombolysis” AND “Pulmonary embolism”. Two independent investigators (PZ and SM), blinded to each other, screened titles and abstracts, with potentially eligible full-text references and subsequently evaluated them against the following eligibility criteria: (1) randomized controlled trials (RCTs), comparing the efficacy or safety of different treatment strategies for patients with PE; (2) observational single- or double-arm study evaluating a treatment strategy for confirmed PE; (3) studies including patients with intermediate-risk (submassive) or high-risk (massive) PE; (4) a treatment strategy of interest including ST, CBT (CDT or MT or both), and AC; (5) exclusion of review articles, case reports, letter to the editors, editorials, and conference abstracts, and studies derived from registries or databases. To ensure completeness, we manually reviewed the reference lists of eligible studies to identify additional relevant studies that might not have been captured in the original database search. For consistency, if discrepancies in PE risk categorization were found, two independent researchers converted the terms massive/submassive to high/intermediate risk according to the European Society of Cardiology guidelines and definitions (PZ, MF) [2]. Any disagreements in study selection were resolved by discussing with a third investigator (DV).

### 2.2. Data Extraction and Quality Assessment

Following the identification of eligible studies, study-level characteristics were extracted independently by two investigators (PZ and SM) and cross-checked blindly with each other, including study location by country, study setting, number of patients, intervention studied, PE severity, and comparative outcome mortality, and major bleeding observed in each study arm. Disagreements were resolved by discussion, and a final decision was reached by consensus with the addition of a third reviewer (DV). For the 11 studies that were included in the meta-analysis, we used appropriate tools to assess the risk of bias. The Cochrane Risk of Bias 2 (RoB 2) tool was utilized for the RCTs, evaluating bias across six domains: (1) randomization process, (2A) deviations from intended interventions (effect of assignment), (2B) deviations from intended interventions (effect of adhering), (3) missing outcome data, (4) measurement of the outcome, and (5) selection of reported results [13]. The ROBINS-I tool was applied for observational studies, assessing bias across seven domains: (1) confounding, (2) classification of interventions, (3) selection of participants, (4) deviations from intended interventions, (5) missing data, (6) measurement of outcomes, and (7) selection of reported results [14]. Assessments were independently performed by two reviewers (MS, MG), with discrepancies resolved through consensus and the involvement of a pair of third reviewers (PS, SS).

### 2.3. Study Outcomes and Statistical Analysis

The primary effectiveness outcome was comparative short-term and midterm all-cause mortality in intermediate-risk PE. Short-term mortality was defined as death of any cause occurring in-hospital or within 30 days. Midterm mortality was defined as death from any cause occurring within 90 days or 1 year, depending on the reported data from the studies. Safety outcomes included minor bleeding, major bleeding, and intracranial hemorrhage (ICH). Major bleeding was defined based on the Bleeding Academic Research Consortium (BARC) classification, where BARC type 3 to 5 was considered as major bleeding, and Global Use of Streptokinase and t-PA for Occluded Coronary Arteries (GUSTO), where ICH or bleeding requiring treatment for hemodynamic compromise was classified as major bleeding. Bleeding events that did not meet the criteria for major bleeding were classified as minor bleeding. We also investigated the crude incidence of mortality and major bleeding for individual interventions in patients with either intermediate or high-risk PE. 

Descriptive statistics are presented as means (± SD) for continuous variables and as number of cases (n) and percentages (%) for dichotomous and categorical variables. Comparative outcomes were assessed using relative risks (RR) obtained from each trial. The cumulative incidence of mortality among patients with PE and the 95% CI was estimated and synthesized using Freeman–Tukey double-arcsine transformations. Random-effects meta-analysis was conducted using the restricted maximum likelihood method as a heterogeneity estimator, given that between-trial variance was inevitable. Heterogeneity was assessed using I^2^ statistics, with estimated values of I^2^ < 50%, 50% < I^2^ < 75%, and I^2^ > 75% indicating low, moderate, and high heterogeneity, respectively. A *p* < 0.05 was considered significant. When the study number for the outcomes of interest was greater than 10, we created funnel plots to assess small-study effects. We subsequently utilized Egger’s test to assess funnel plot asymmetry, with *p* < 0.05 indicating significant asymmetry. The analyses were performed using STATA software (version 14.1; StataCorp College Station, TX, USA), R Studio 2024.04.2-764, and OpenMEE (OpenMeta [Analyst]) 5.12.14.

## 3. Results

### 3.1. Search Results 

**Figure 1:**
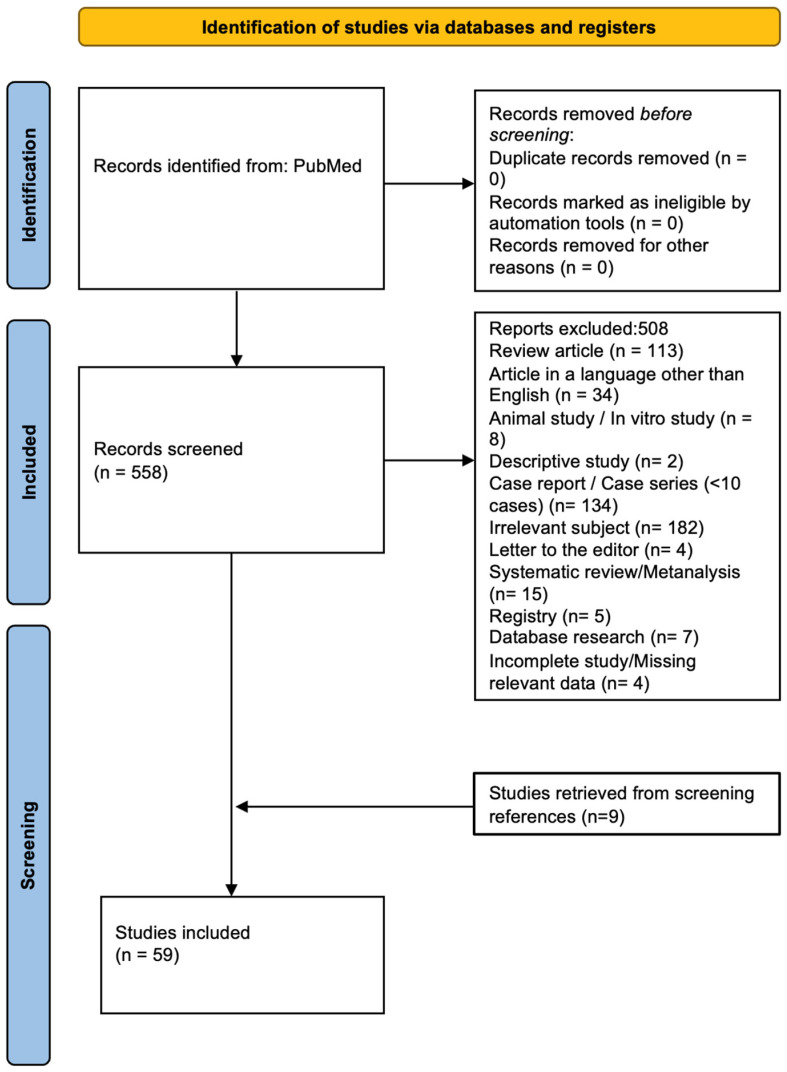
PRISMA flowchart.

The literature search yielded 558 potentially eligible studies. A total of 507 studies were excluded, as shown in the PRISMA flowchart (Figure 1). Ultimately, 59 studies (Table 1) fulfilled all the predetermined inclusion criteria and were included in our meta-analysis.

### 3.2. Study and Patient Characteristics (PE Severity, Mortality, Bleeding) 

**Figure 2:**
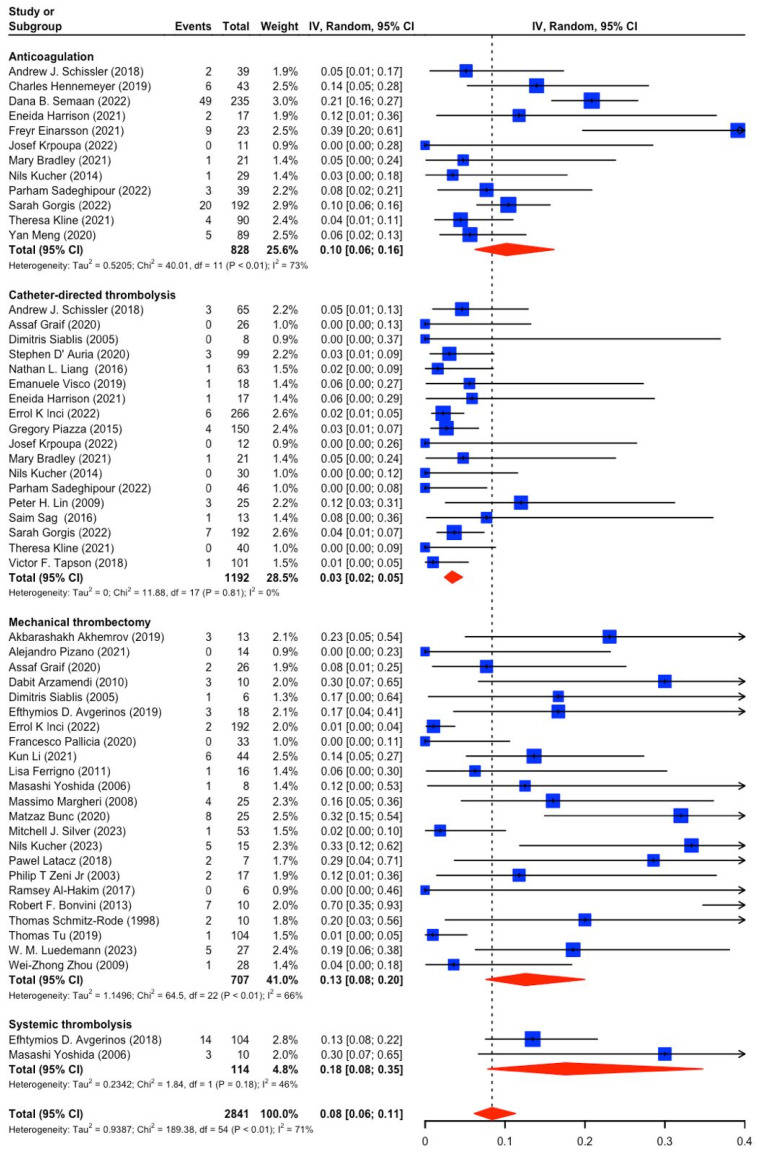
Cumulative mortality among included studies [11,15,16,17,18,19,20,21,22,23,24,25,26,28,30,31,32,33,37,38,39,42,43,48,49,50,51,52,53,55,57,58,60,61,62,63,64,65,66,67,68,70,72].

Data were collected from 59 studies, including 4457 patients in our analysis. For the initial prevalence study, we included only those studies where patients were treated only with a distinct intervention, excluding the studies combining MT and CDT [11,15,16,17,18,19,20,21,22,23,24,25,26,27,28,29,30,31,32,33,34,35,36,37,38,39,40,41,42,43,44,45,46,47,48,49,50,51,52,53,54,55,56,57,58,59,60,61,62,63,64,65,66,67,68,69,70,71,72]. For the initial prevalence study, the cumulative mortality in this population was 8% (95% CI [6–11%]), with a total of 211 events in 2841 patients (Figure 2). Among the analyzed treatment modalities, CDT was the safest, with a mortality of 3% (95% CI [2–5%]), followed by AC, with a mortality of 10% (95% CI [6–16%]). MT and ST were among the treatments with higher mortality of 13% (95% CI [8–20%]) and 18% (95% CI [8–35%]), respectively.

**Figure 3:**
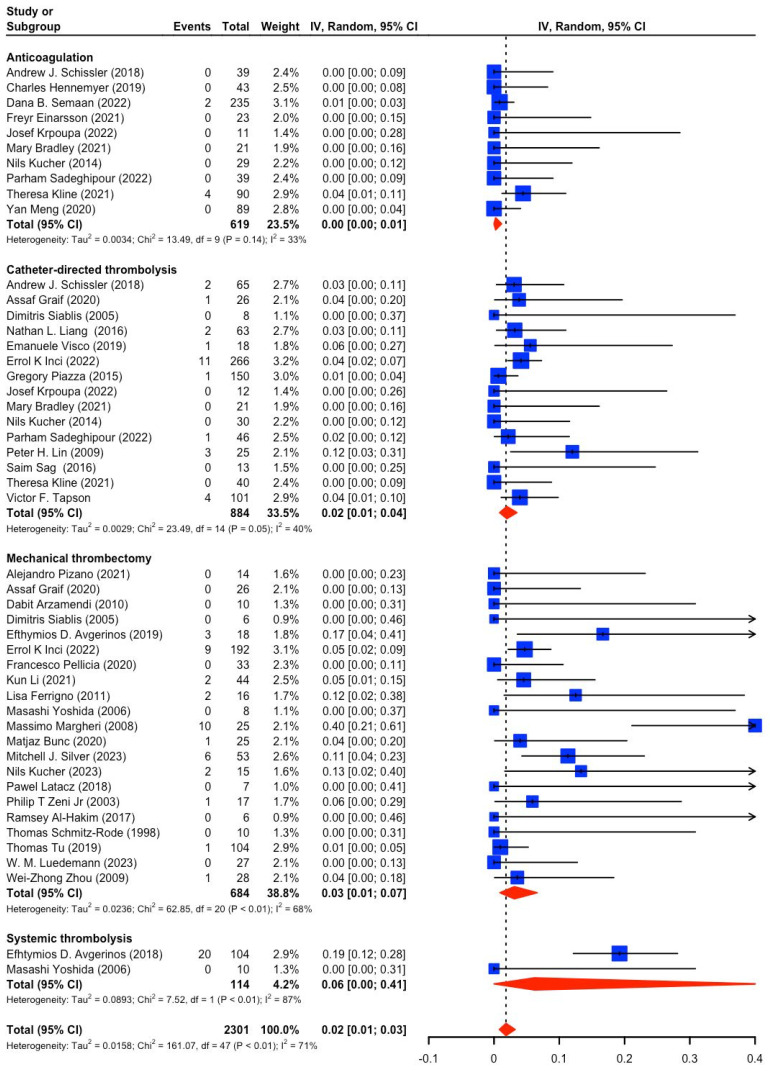
Cumulative incidence of bleeding among included studies [15,16,17,18,19,20,21,22,23,24,25,28,30,31,32,37,38,39,40,42,43,48,50,51,52,53,55,57,58,60,61,63,64,65,67,70,72].

The cumulative incidence of major bleeding in the overall population was 2% [1–3%], with a total of 90 events among 2301 patients with available data (Figure 3). 

### 3.3. Catheter-Based Therapies (CBT) vs. Anticoagulation (AC) in Intermediate-Risk PE

**Figure 4:**
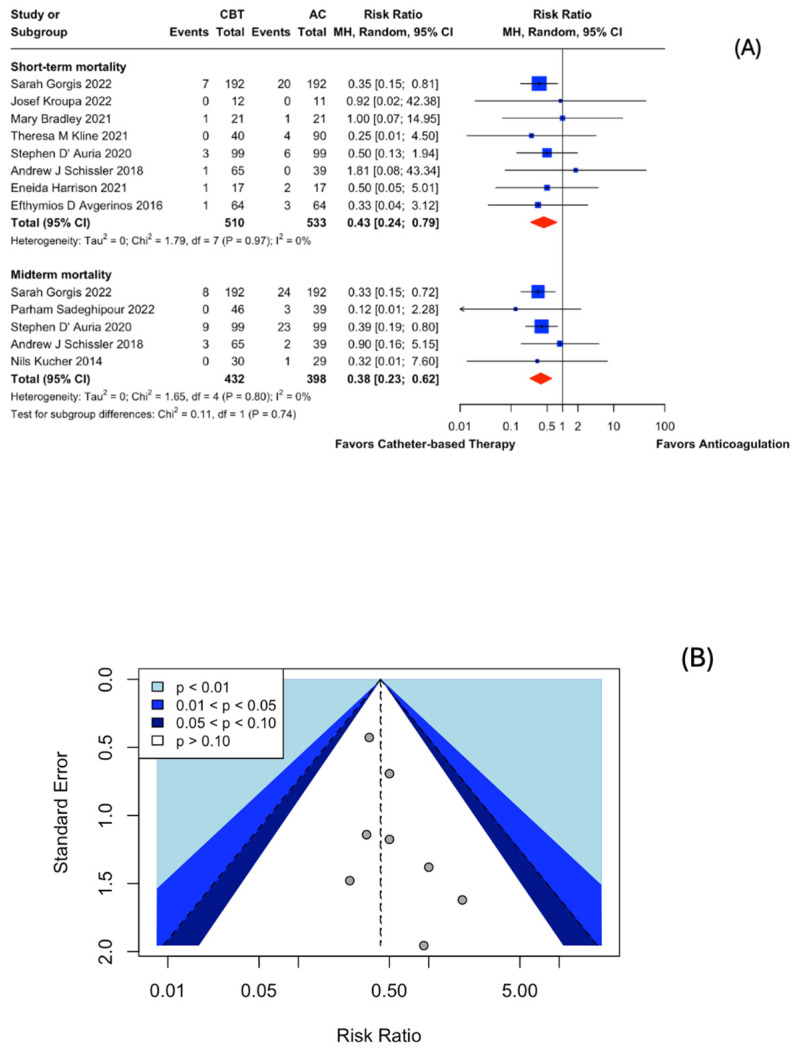
(**A**) A forest plot for comparative short-term and midterm all-cause mortality of CBT vs. AC. (**B**) A funnel plot for comparative short-term mortality between CBT and AC [11,22,62,63,64,65,66,67,68,69].

Out of the 59 studies included a total of 11 studies, including 3 RCTs, were used to compare the effectiveness and safety between CBT and AC in patients with intermediate-risk PE. The use of CBT was associated with a significant 57% reduction in short-term all-cause mortality (RR, 0.43; 95% CI, 0.24 to 0.79; I^2^ = 0%; 95% CI, 0.0% to 67.6%) (Figure 4A) when compared with AC, with no statistical heterogeneity. For midterm all-cause mortality, CBT use was also associated with significantly lower all-cause death than AC (RR, 0.38; 95% CI, 0.23 to 0.62; I^2^ = 0%; 95% CI, 0.0% to 67.6%) (Figure 4A), with no statistical heterogeneity. Funnel plot with Egger’s test for short-term mortality did not show evidence of asymmetry (*p* = 0.78) (Figure 4B), indicating no evidence of small-study effects.

**Figure 5:**
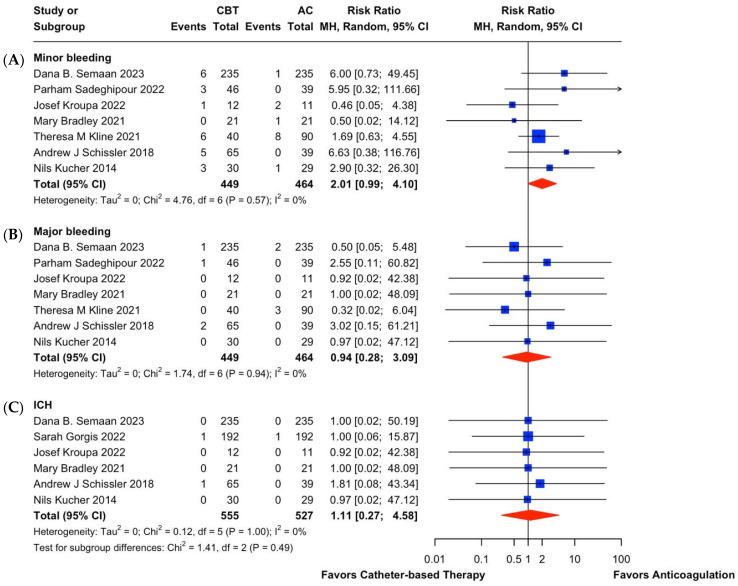
A forest plot for comparative (**A**) minor bleeding, (**B**) major bleeding and (**C**) ICH between CBT vs. AC [11,22,43,62,63,64,65,67].

With regards to the safety outcomes (Figure 5), there was no statistically significant difference in bleeding between CBT and AC. Nonetheless, the difference in minor bleeding was borderline insignificant (RR, 2.01; 95% CI, 0.99 to 4.10; I^2^ = 0%; 95% CI, 0.0% to 67.6%), with a trend favoring AC over CBT. Major bleeding (RR, 0.94; 95% CI, 0.28 to 3.09; I^2^ = 0%; 95% CI, 0.0% to 70.8%), and ICH (RR, 1.11; 95% CI, 0.27 to 4.58; I^2^ = 0%; 95% CI, 0.0% to 67.6%) did not show any statistically significant difference between CBT and AC.

### 3.4. Quality (Risk of Bias) Assessment

**Figure 6:**
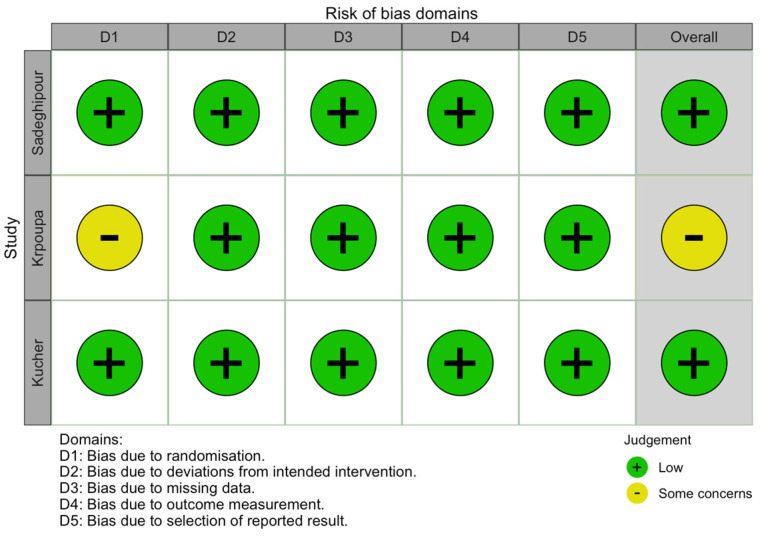
Risk of bias for RCTs using RoB2 [11,63,64].

**Figure 7:**
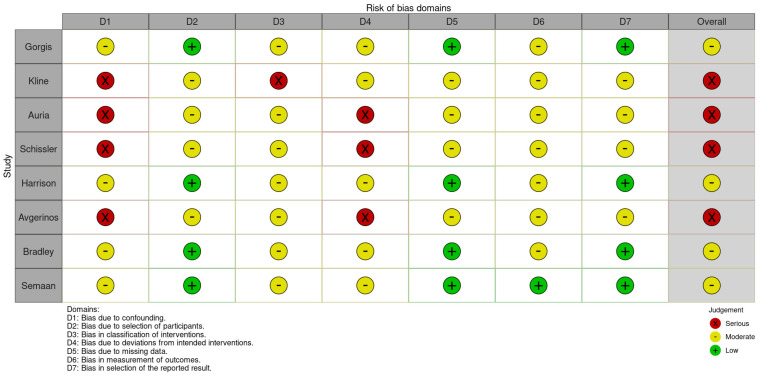
Risk of bias for observational studies using ROBINS-V2 [22,43,62,65,66,67,68,69].

Among the RCTs, the overall risk of bias was low in two studies (Parham Sadeghipour et al., 2022 and Nils Kucher et al., 2014) [11,63] and raised some concerns in one study (Josef Kroupa et al., 2022) [64], mainly due to insufficient clarity in the description of the randomization concealment procedure (Figure 6). For the observational studies, four studies had an overall moderate risk of bias (Sarah Gorgis et al., 2022; Eneida Harrison et al., 2021; Mary Bradley et al., 2021; Dana B. Semaan et al., 2022) [22,43,62,68]. The moderate rating was primarily related to partial control of confounding factors and moderate deviations from intended interventions. The remaining four observational studies (Theresa Kline et al., 2021; Stephen D’Auria et al., 2020; Andrew J. Schissler et al., 2018; Efthymios D. Avgerinos et al., 2016) [65,66,67,69] were assessed as having a serious risk of bias, predominantly due to inadequate adjustment for confounding variables, significant deviations due to a lack of standardized intervention protocols, and elevated potential for selection bias (Figure 7). 

## 4. Discussion

The pathophysiologic cascade during acute PE can lead to circulatory collapse and RV overload, which is considered to be the primary cause of death. Once the emboli cover a certain cross-sectional area of the pulmonary vasculature, there is a pulmonary artery pressure (PAP) increase that is further exacerbated by physiologic hypoxic vasoconstriction that aims to reverse the V/Q mismatch [73,74]. The sudden increase in PAP cannot be overcome by a thin-walled RV, which eventually leads to decreased LV filling and reduced cardiac output, a mechanism that is further exacerbated by the neurohormonal cascade that occurs [3,75,76]. CBTs have been shown to improve RV hemodynamics, showing promise in treating patients with RV dysfunction. For example, several thrombectomy devices have demonstrated efficacy with regard to RV hemodynamics in patients with intermediate to high-risk PE leading to a decrease in RV/LV ratio, sPAP, and mPAP [12,77,78]. Similarly, ultrasound-assisted catheter-directed thrombolysis (USCDT) has also shown promising results with regard to RV hemodynamics, leading to a reversal of RV dilation within 24 h [11].

In our study, we further analyzed the data based on the severity of PE. We found 11 studies that directly compared CBT to AC for intermediate-risk PE, and we found that treatment with CBT instead of AC alone was associated with improved outcomes both in terms of short-term mortality and midterm mortality. Interestingly, a network meta-analysis performed by Planer et al. found improved all-cause mortality in patients treated with CDT compared to ST (OR 0.43 [0.32–0.57]) [79]. In the same study, CDT was compared to AC and CDT was again proven to be superior, [79] a finding that is consistent with our findings as well, but our findings from the meta-analysis focus on intermediate-risk pulmonary embolism.

Our findings support the hypothesis that improved RV function leads to lower mortality [80]. Additional studies will be needed to solidify this finding, and currently, there are several ongoing trials measuring the efficacy of USCDT versus AC and MT versus AC, which will provide further data for the use of CBTs (BETULA (NCT03854266), STRATIFY (NCT04088292), HI-PEITHO (NCT04790370), PEERLESS II) [81,82]. Another recent study, PEERLESS, comparing CDT to MT for intermediate-risk PE sheds light as to which catheter intervention provides the best outcomes, as it is the first prospective study to compare the two. It appears that there is no significant difference in all-cause mortality, but patients treated with MT appear to have reduced ICU admission, 30-day readmission rate, and reduced hospital stay [83]. 

Another benefit of CBTs is the reduced risk of major bleeding, especially in comparison to ST. Bleeding is one of the major complications of most therapies in the treatment of pulmonary embolism. Major bleeding has been identified as one of the independent predictors of in-hospital and 90-day mortality, according to the results from the prospective ZATPOL registry [84]. Traditionally, systemic thrombolysis has been proposed to have a higher bleeding risk as compared to newer treatment strategies, with figures suggesting around a 10% rate of major bleeding and a 3–5% risk of intracranial hemorrhage [85]. The Pulmonary Embolism International Thrombolysis (PEITHO) trial studied outcomes in 1006 patients with intermediate-risk pulmonary embolism undergoing systemic thrombolysis using Tenecteplase with standard anticoagulation [86]. Patients undergoing systemic thrombolysis had an increased risk of both major bleeding (6.3 vs. 1.5%, *p* < 0.001) and intracranial hemorrhage (2.4% vs. 0.2%, *p* < 0.001) in comparison to the anticoagulation arm [86]. Similar results were also seen in a meta-analysis by Chatterjee et.al which included 2115 patients [87]. They found a higher rate of major bleeding (9.24% vs. 3.42%, OR, 2.73; 95% CI, 1.91–3.91) and intracranial hemorrhage (1.46% vs. 0.19%, OR, 4.63; 95% CI, 1.78–12.04) in patients receiving systemic thrombolysis as compared to anticoagulation alone. Notably, the difference in major bleeding rates was apparent only in patients older than 65 years. From the prevalence analysis in our study, major bleeding with AC appears to be less common than in patients receiving ST, but this is not a direct comparison, and the number of ST studies is limited. 

Despite being proposed as a rescue treatment for high-risk PE in cases where systemic thrombolysis failed or was contraindicated (class of recommendation IIa, level of evidence C) in the 2019 guidelines by the European Society of Cardiology (ESC), CBTs are increasingly being used for management of acute PE especially given a lower bleeding risk [3]. In our study, the prevalence of major bleeding in patients receiving either CDT or MT was lower than in patients receiving ST, a finding that is consistent with a prior meta-analysis by Pietrasik et al. comparing CBTs (which included intrapulmonary administration of low doses of thrombolytic drugs, ultrasound-assisted thrombolysis, mechanical aspiration thrombectomy, and direct clot retrieval systems) to systemic thrombolysis [85]. Pietrasik et al. also reported that while bleeding complications were more frequent in the CBT group, the rates of major bleeding, hemoptysis, and the need for RBC transfusion in patients were significantly lower (11.9 vs. 17.4% for major bleeding, 2.7 vs. 1.1% for hemoptysis, and 9.6 vs. 14.6% for RBC transfusion; *p* ≤ 0.01 for all) compared to ST [85]. 

Monteleone et al. in the REAL-PE study examined outcomes in 2259 patients with PE, out of whom 1577 received ultrasound-assisted catheter-directed thrombolysis (USCDT) and 682 patients received MT using the FlowTriever system (Inari Medical, Irvine, CA, USA) [88]. They also performed a contemporary analysis involving 1798 patients, wherein 1137 received USCDT and 661 underwent MT. The results consistently showed a higher incidence of bleeding in patients who underwent MT compared to US CDT as evidenced by higher rates of decrease in hemoglobin levels by more than 2 g/dL (67.4% vs. 53.4%; *p* < 0.0001) and decrease in hemoglobin levels by more than 5 g/dL (22.6% vs. 14.8%; *p* < 0.0001). Patients undergoing MT also had a greater requirement for blood transfusion (5.9% vs. 1.9%; *p* < 0.0001) and major bleeding as defined by the International Society for Thrombosis and Hemostasis (ISTH) (17.3% vs. 12.4%; *p* = 0.0018) and Bleeding Academic Research Consortium (BARC) (15.4% vs. 11.8%; *p* = 0.019) criteria. In contrast, a multicenter retrospective cohort study directly comparing CDT to MT including 458 patients with acute intermediate or high-risk PE who either received CDT (n = 266) or underwent MT (n = 192) did not find any difference in major bleeding between the two treatment arms (4.2% in CDT group vs. 4.7% in the MT group; *p* = 0.8)

The lower risk of bleeding in CBT compared to ST, could be attributed to the advantage of targeted therapy directly into the pulmonary clot, which involves considerably lower doses of thrombolytics compared to ST. Improved surgical techniques, especially those pertaining to closure of the puncture wound for large-bore mechanical devices, have substantially helped lower the chances of bleeding, and smaller sheath sizes used for CDT, along with very low thrombolytic doses, have also contributed to the lower bleeding risks. When comparing CBT to AC we did not find a statistically significant difference in major bleeding events in patients with intermediate-risk PE. We also found that the prevalence of major bleeding in CDT and MT appears to be similar between the two groups, but this was not a direct comparison of the two treatment modalities.

## 5. Limitations

Several limitations should be considered when interpreting the results. First, the majority of the included studies in our study were observational, which renders our study results as hypothesis-generating in nature, inherently limiting the conclusion around causal inference. Second, while there was no statistical heterogeneity being observed in our results, conceptual heterogeneity cannot be excluded. In particular, the lack of specific guidelines and robust data regarding catheter-based therapies is a major reason protocols differ from study to study. We did not account for the severity of pulmonary embolism in the prevalence analysis, but we did so in the meta-analysis, comparing catheter-based therapies to anticoagulation. Even though patients were classified based on the PE severity, the lack of specific indications for the use of CBTs meant that each study and institution may have had different inclusion criteria, and in some cases, CBTs may have been preferentially given to overall healthier patients, which can potentially introduce indication bias skewing the results in favor of CBTs. Similarly, for bleeding we wanted to ensure that major bleeding was accounted for in our study, leading to some studies being excluded as they did not specify the severity of bleeding or did not classify bleeding events based on widely accepted classification systems such as BARC, GUSTO, ISTH, etc. Third, given the lack of large clinical trials on CBTs, there was heterogeneity in the techniques that were used as well as the dosages of infused medications (e.g., TPA). Lastly, to increase the number of included studies, we had to analyze RCTs and observational studies together. As anticipated, while RCT evidence generally provides robust conclusions regarding catheter-based therapies versus anticoagulation for intermediate-risk pulmonary embolism, the observational studies present inherent methodological limitations, necessitating cautious interpretation. Further large-scale, methodologically rigorous randomized studies are warranted to confirm these findings.

## 6. Conclusions

Our study found that CBT was associated with a significantly lower risk of all cause mortality when compared to AC alone in patients with intermediate-risk PE. Importantly, the risk of major bleeding remained comparable between the two interventions. Ongoing RCTs will shed more light on the role of CBT in PE management and help identify patient populations that may derive the greatest benefit from these interventions. 

## Figures and Tables

**Table 1:** Included studies characteristics [11,15,16,17,18,19,20,21,22,23,24,25,26,27,28,29,30,31,32,33,34,35,36,37,38,39,40,41,42,43,44,45,46,47,48,49,50,51,52,53,54,55,56,57,58,59,60,61,62,63,64,65,66,67,68,69,70,71].

First Author (Year)	Country	Total N	Age, Mean (SD)	Sex (M/F)	Arm 1	Arm 2	PE Severitiy
Nils Kucher (2014)	Multinational	59	63 (14)	28/31	CDT	AC	Intermediate-risk
Errol K. Inci (2022)	USA	458	57 (1.67)	217/241	MT	CDT	Intermediate-risk/High-risk
Efthymios D. Avgerinos (2019)	USA	72	63.6 (15)	31/41	CDT	MT	Intermediate-risk/High-risk
Matjaz Bunc (2020)	Slovenia		62.6 (12.7)	16/9	MT	NA	High-risk
Francesco Pelliccia (2020)	Italy	33	43 (13)	20/13	MT	NA	High-risk
Nils Kucher (2023)	Switzerland	15	63 (12)	14/1	MT	NA	High-risk
Assaf Graif (2020)	USA	52	59.7 (20.9)	24/28	MT	CDT	Intermediate-risk
Alejandro Pizano (2021)	USA	14	60 (median)	9/5	MT	NA	Intermediate-risk
Mary Bradley (2021)	USA	42	56.8 (24.7)	25/17	CDT	AC	Intermediate-risk
Kun Li (2021)	China	23	58.6 (12.2)	11/12	MT	NA	Intermediate-risk
Kun Li (2021)	China	21	58 (15)	10/11	MT	NA	High-risk
Yan Meng (2020)	China	186	60.3 (12.6)	90/96	CBT	AC	Intermediate-risk/High-risk
Miguel Angel De Gregorio (2019)	Spain	54	59.7 (16.8)	23/31	CBT	NA	High-risk
Pawel Latacz (2018)	Poland	7	52.7 (16.6)	4/3	MT	NA	Intermediate-risk/High risk
Nathan L. Liang (2016)	USA	63	59 (19.2)	27/36	CDT	NA	Intermediate-risk/High-risk
Bing Liu (2018)	China	20	61.4 (13.5)	7/13	CBT	NA	NA
W. M. Luedemann (2023)	Germany	27	56.1 (15.3)	12/15	MT	NA	Intermediate-risk/High-risk
Sheng Liu (2010)	China	14	55.4	8/6	CBT	NA	Intermediate-risk/High-risk
Dimitris Siablis (2005)	Greece	14	63.6 (15.6)	7/7	CDT	MT	NA
Massimo Margheri (2008)	Italy	17	64.2 (13.9)	11/6	MT	NA	Intermediate-risk
Massimo Margheri (2008)	Italy	8	67.4 (11.9)	5/3	MT	NA	High-risk
Mitchell J. Silver (2023)	USA	114	63 (15)	53/61	MT	ST/AC	High-risk
Robert F. Bonvini (2013)	Switzerland	10	73 (9)	5/5	MT	NA	High-risk
Maofeng Gong (2021)	China	48	59.9 (12.3)	29/19	CBT	NA	High-risk
S. Muller-Hulsbeck (2001)	Germany	9	55	4/5	CBT	NA	Intermediate-risk
Jesus Ribas (2021)	Spain	20	57.3 (12.4)	10/10	CBT	NA	Intermediate-risk
Jesus Ribas (2021)	Spain	43	61.6 (14.5)	22/21	CBT	NA	High-risk
Ramsey Al-Hakim (2017)	USA	6	62.7 (19)	3/3	MT	NA	Intermediate-risk
Emanuele Visco (2019)	Italy	18	74 (12.7)	5/13	CDT	NA	Intermediate-risk/High-risk
Charles Hennemeyer (2019)	USA	79	59.5 (18.5)	37/42	CBT	AC	Intermediate-risk/High-risk
Thomas Tu (2019)	USA	104	55.6 (13.7)	56/48	MT	NA	Intermediate-risk
Zoltan Ruzsa (2020)	Hungary	80	59 (16.8)	42/38	CBT	NA	Intermediate-risk
Manish Chauhan (2007)	USA	6	57.8 (12.6)	2/4	CBT	NA	High-risk
Manish Chauhan (2007)	USA	8	67.6 (7.8)	5/3	CBT	NA	Intermediate-risk
Dabit Arzamendi (2010)	Canada	10	43.7 (18.8)	3/7	MT	NA	High-risk
Dana B. Semaan (2022)	USA	470	59.4	244/226	CBT	AC	Intermediate-risk
Takeshi Yamamoto (2008)	Japan	50	62 (15)	19/31	CBT	NA	High-risk
Kazuhiro Nakazawa (2008)	Japan	25	60 (15)	8/17	CBT	NA	High-risk
Max Andresen (2012)	Chile	14	67 (19)	5/9	CBT	NA	Intermediate-risk
Xiangdong Meng (2022)	China	159	61.4 (10.5)	71/88	CBT	NA	Intermediate-risk/High-risk
Thomas Schmitz-Rode (1998)	Germany	10	53.8 (9.5)	6/4	MT	NA	High-risk
Akbarashakh Akhmerov (2019)	USA	13	56 (15)	10/3	MT	NA	NA
Saim Sag (2016)	Turkey	13	51.6 (18.2)	6/7	CDT	NA	High-risk
Philip T Zeni Jr (2003)	USA	17	51.7 (16.6)	9/8	MT	NA	High-risk
Freyr Einarsson (2021)	Sweden	45	70,68 (median for each group)	20/25	CBT	AC	High-risk
Efhtymios D. Avgerinos (2018)	USA	90	58.8 (15.8)	152/165	CBT	ST	High-risk
Efhtymios D. Avgerinos (2018)	USA	227	58.8 (15.8)	152/165	CBT	ST	Intermediate-risk
Lisa Ferrigno (2011)	USA	5	54 (16.4)	3/2	MT	NA	High-risk
Lisa Ferrigno (2011)	USA	11	54.2 (13.5)	4/7	MT	NA	Intermediate-risk
Laurencia Villalba (2019)	Australia	32	65.8	17/15	CBT	NA	Intermediate-risk/High-risk
Peter H. Lin (2009)	USA	25	60.7 (29.6)	12/13	CDT	NA	Intermediate-risk/High-risk
Nathan L. Liang (2017)	USA	69	59.2 (15.4)	30/39	CBT	NA	Intermediate-risk/High-risk
Wei-Zhong Zhou (2009)	China	28	63.5 (11.5)	20/8	MT	NA	Intermediate-risk/High-risk
Masashi Yoshida (2006)	Japan	18	64.3 (25.5)	7/11	MT	ST	NA
J. Hubbard (2011)	USA	11	60.2	9/2	CBT	NA	Intermediate-risk/High-risk
Gregory Piazza (2015)	USA	150	59 (16.1)	73/77	CDT	NA	Intermediate-risk/High-risk
Victor F. Tapson (2018)	Multinational	101	60 (median)	53/48	CDT	NA	Intermediate-risk
Sarah Gorgis (2022)	Italy	384	59.3 (15.1)	188/196	CDT	AC	Intermediate-risk
Parham Sadeghipour (2022)	Iran	94	57.6 (2.4)	61/24	CDT	AC	Intermediate-risk
Josef Kroupa (2022)	Czech Republic	23	61.9 (25.6)	13/10	CDT	AC	Intermediate-risk
Theresa Kline (2021)	USA	130	63 (median)	57/73	CDT	AC	Intermediate-risk
Stephen D’ Auria (2020)	USA	198	NA	103/95	CDT	AC	Intermediate-risk
Andrew J. Schissler (2018)	USA	104	55.5 (16.7)	46/58	CDT	AC	Intermediate-risk
Eneida Harrison (2021)	USA	34	75.6 (11)	NA	CDT	AC	Intermediate-risk
Efthymios D. Avgerinos (2016)	USA	128	59.3 (16.7)	63/65	CBT	AC	Intermediate-risk

N—number of patients in the study, SD—standard deviation, M—male, F—female, PE—pulmonary embolism. Studies in blue contrained separate data for intermediate and high-risk PE [11,15,16,17,18,19,20,21,22,23,24,25,26,27,28,29,30,31,32,33,34,35,36,37,38,39,40,41,42,43,44,45,46,47,48,49,50,51,52,53,54,55,56,57,58,59,60,61,62,63,64,65,66,67,68,69,70,71].

## Data Availability

No new data were created for this study. All data derived from published studies.

## References

[1] Jaff M.R., McMurtry M.S., Archer S.L., Cushman M., Goldenberg N., Goldhaber S.Z., Jenkins J.S., Kline J.A., Michaels A.D., Thistlethwaite P. (2011). Management of Massive and Submassive Pulmonary Embolism, Iliofemoral Deep Vein Thrombosis, and Chronic Thromboembolic Pulmonary Hypertension. Circulation.

[2] Konstantinides S. (2014). V 2014 ESC Guidelines on the Diagnosis and Management of Acute Pulmonary Embolism. Eur. Heart J..

[3] Konstantinides S.V., Meyer G., Becattini C., Bueno H., Geersing G.-J., Harjola V.-P., Huisman M.V., Humbert M., Jennings C.S., Jiménez D. (2020). 2019 ESC Guidelines for the Diagnosis and Management of Acute Pulmonary Embolism Developed in Collaboration with the European Respiratory Society (ERS). Eur. Heart J..

[4] Kuo W.T., Sista A.K., Faintuch S., Dariushnia S.R., Baerlocher M.O., Lookstein R.A., Haskal Z.J., Nikolic B., Gemmete J.J. (2018). Society of Interventional Radiology Position Statement on Catheter-Directed Therapy for Acute Pulmonary Embolism. J. Vasc. Interv. Radiol..

[5] Rivera-Lebron B.N., Rali P.M., Tapson V.F. (2021). The PERT Concept. Chest.

[6] Stevens S.M., Woller S.C., Kreuziger L.B., Bounameaux H., Doerschug K., Geersing G.-J., Huisman M.V., Kearon C., King C.S., Knighton A.J. (2021). Antithrombotic Therapy for VTE Disease. Chest.

[7] Giri J., Sista A.K., Weinberg I., Kearon C., Kumbhani D.J., Desai N.D., Piazza G., Gladwin M.T., Chatterjee S., Kobayashi T. (2019). Interventional Therapies for Acute Pulmonary Embolism: Current Status and Principles for the Development of Novel Evidence: A Scientific Statement From the American Heart Association. Circulation.

[8] Frat J.-P., Ciurzyński M. (2024). Intermediate-Risk Acute Pulmonary Embolism. Chest.

[9] Chen Y.L., Wright C., Pietropaoli A.P., Elbadawi A., Delehanty J., Barrus B., Gosev I., Trawick D., Patel D., Cameron S.J. (2020). Right Ventricular Dysfunction Is Superior and Sufficient for Risk Stratification by a Pulmonary Embolism Response Team. J. Thromb. Thrombolysis.

[10] Schoepf U.J., Kucher N., Kipfmueller F., Quiroz R., Costello P., Goldhaber S.Z. (2004). Right Ventricular Enlargement on Chest Computed Tomography: A Predictor of Early Death in Acute Pulmonary Embolism. Circulation.

[11] Kucher N., Boekstegers P., Müller O.J., Kupatt C., Beyer-Westendorf J., Heitzer T., Tebbe U., Horstkotte J., Müller R., Blessing E. (2014). Randomized, Controlled Trial of Ultrasound-Assisted Catheter-Directed Thrombolysis for Acute Intermediate-Risk Pulmonary Embolism. Circulation.

[12] Lauder L., Pérez Navarro P., Götzinger F., Ewen S., Al Ghorani H., Haring B., Lepper P.M., Kulenthiran S., Böhm M., Link A. (2023). Mechanical Thrombectomy in Intermediate- and High-Risk Acute Pulmonary Embolism: Hemodynamic Outcomes at Three Months. Respir. Res..

[13] Sterne J.A.C., Savović J., Page M.J., Elbers R.G., Blencowe N.S., Boutron I., Cates C.J., Cheng H.-Y., Corbett M.S., Eldridge S.M. (2019). RoB 2: A Revised Tool for Assessing Risk of Bias in Randomised Trials. BMJ.

[14] Sterne J.A., Hernán M.A., Reeves B.C., Savović J., Berkman N.D., Viswanathan M., Henry D., Altman D.G., Ansari M.T., Boutron I. (2016). ROBINS-I: A Tool for Assessing Risk of Bias in Non-Randomised Studies of Interventions. BMJ.

[15] Inci E.K., Khandhar S., Toma C., Licitra G., Brown M.J., Herzig M., Matthai W., Palevsky H., Schwartz A., Wight J.A. (2023). Mechanical Thrombectomy versus Catheter Directed Thrombolysis in Patients with Pulmonary Embolism: A Multicenter Experience. Catheter. Cardiovasc. Interv..

[16] Avgerinos E.D., Abou Ali A., Toma C., Wu B., Saadeddin Z., McDaniel B., Al-Khoury G., Chaer R.A. (2019). Catheter-Directed Thrombolysis versus Suction Thrombectomy in the Management of Acute Pulmonary Embolism. J. Vasc. Surg. Venous Lymphat. Disord..

[17] Bunc M., Steblovnik K., Zorman S., Popovic P. (2020). Percutaneous Mechanical Thrombectomy in Patients with High-Risk Pulmonary Embolism and Contraindications for Thrombolytic Therapy. Radiol. Oncol..

[18] Pelliccia F., De Luca A., Pasceri V., Tanzilli G., Speciale G., Gaudio C. (2020). Safety and Outcome of Rheolytic Thrombectomy for the Treatment of Acute Massive Pulmonary Embolism. J. Invasive Cardiol..

[19] Kucher N., Ouda A., Voci D., Barco S., Micieli E., Münger M., Pleming W., Grigorean A., Sromicki J., Schmiady M.O. (2023). Percutaneous Large-Bore Aspiration Embolectomy with Veno-Arterial Extracorporal Membrane Oxygenation Support or Standby in Patients with High-Risk Pulmonary Embolism and Contraindications to Thrombolysis: A Preliminary Single Centre Experience. Eur. Heart J. Acute Cardiovasc. Care.

[20] Graif A., Patel K.D., Wimmer N.J., Kimbiris G., Grilli C.J., Upparapalli D., Kaneria A.R., Leung D.A. (2020). Large-Bore Aspiration Thrombectomy versus Catheter-Directed Thrombolysis for Acute Pulmonary Embolism: A Propensity Score-Matched Comparison. J. Vasc. Interv. Radiol..

[21] Pizano A., Ray H.M., Cambiaghi T., Saqib N.U., Afifi R., Khan S., Martin G., Harlin S.A. (2022). Initial Experience and Early Outcomes of the Management of Acute Pulmonary Embolism Using the FlowTriever Mechanical Thrombectomy Device. J. Cardiovasc. Surg..

[22] Bradley M., Bull T., Hountras P., MacLaren R. (2022). Pragmatic Use of Catheter-Directed Thrombolysis in Venous Thromboembolism and a Comparative Evaluation With Traditional Therapies in Submassive Pulmonary Embolism. J. Pharm. Pract..

[23] Li K., Cui M., Zhang K., Liang K., Liu H., Zhai S. (2021). Treatment of Acute Pulmonary Embolism Using Rheolytic Thrombectomy. EuroIntervention.

[24] Meng Y., Zhang J., Ma Q., Qin H., Zhang B., Pang H., Yin Q., Tian H. (2020). Pulmonary Interventional Therapy for Acute Massive and Submassive Pulmonary Embolism in Cases Where Thrombolysis Is Contraindicated. Ann. Vasc. Surg..

[25] Latacz P., Simka M., Brzegowy P., Serednicki W., Konduracka E., Mrowiecki W., Słowik A., Łasocha B., Mrowiecki T., Popiela T. (2018). Treatment of High- and Intermediate-Risk Pulmonary Embolism Using the AngioJet Percutaneous Mechanical Thrombectomy System in Patients with Contraindications for Thrombolytic Treatment—A Pilot Study. Wideochir Inne Tech. Maloinwazyjne.

[26] Liang N.L., Avgerinos E.D., Marone L.K., Singh M.J., Makaroun M.S., Chaer R.A. (2016). Comparative Outcomes of Ultrasound-Assisted Thrombolysis and Standard Catheter-Directed Thrombolysis in the Treatment of Acute Pulmonary Embolism. Vasc. Endovasc. Surg..

[27] Liu B., Liu M., Yan L., Yan J., Wu J., Jiao X., Guo M. (2018). Percutaneous Mechanical Thrombectomy Combined with Catheter-Directed Thrombolysis in the Treatment of Acute Pulmonary Embolism and Lower Extremity Deep Venous Thrombosis: A Novel One-Stop Endovascular Strategy. J. Int. Med. Res..

[28] Luedemann W.M., Zickler D., Kruse J., Koerner R., Lenk J., Erxleben C., Torsello G.F., Fehrenbach U., Jonczyk M., Guenther R.W. (2023). Percutaneous Large-Bore Pulmonary Thrombectomy with the FlowTriever Device: Initial Experience in Intermediate-High and High-Risk Patients. Cardiovasc. Interv. Radiol..

[29] Liu S., Shi H.-B., Gu J.-P., Yang Z.-Q., Chen L., Lou W.-S., He X., Zhou W.-Z., Zhou C.-G., Zhao L.-B. (2011). Massive Pulmonary Embolism: Treatment with the Rotarex Thrombectomy System. Cardiovasc. Interv. Radiol..

[30] Siablis D., Karnabatidis D., Katsanos K., Kagadis G.C., Zabakis P., Hahalis G. (2005). AngioJet Rheolytic Thrombectomy versus Local Intrapulmonary Thrombolysis in Massive Pulmonary Embolism: A Retrospective Data Analysis. J. Endovasc. Ther..

[31] Margheri M., Vittori G., Vecchio S., Chechi T., Falchetti E., Spaziani G., Giuliani G., Rovelli S., Consoli L., Biondi Zoccai G.G.L. (2008). Early and Long-Term Clinical Results of AngioJet Rheolytic Thrombectomy in Patients with Acute Pulmonary Embolism. Am. J. Cardiol..

[32] Silver M.J., Gibson C.M., Giri J., Khandhar S., Jaber W., Toma C., Mina B., Bowers T., Greenspon L., Kado H. (2023). Outcomes in High-Risk Pulmonary Embolism Patients Undergoing FlowTriever Mechanical Thrombectomy or Other Contemporary Therapies: Results From the FLAME Study. Circ. Cardiovasc. Interv..

[33] Bonvini R.F., Roffi M., Bounameaux H., Noble S., Müller H., Keller P.-F., Jolliet P., Sarasin F.P., Rutschmann O.T., Bendjelid K. (2013). AngioJet Rheolytic Thrombectomy in Patients Presenting with High-Risk Pulmonary Embolism and Cardiogenic Shock: A Feasibility Pilot Study. EuroIntervention.

[34] Gong M., Chen G., Zhao B., Kong J., Gu J., He X. (2021). Rescue Catheter-Based Therapies for the Treatment of Acute Massive Pulmonary Embolism after Unsuccessful Systemic Thrombolysis. J. Thromb. Thrombolysis.

[35] Müller-Hülsbeck S., Brossmann J., Jahnke T., Grimm J., Reuter M., Bewig B., Heller M. (2001). Mechanical Thrombectomy of Major and Massive Pulmonary Embolism with Use of the Amplatz Thrombectomy Device. Investig. Radiol..

[36] Ribas J., Valcárcel J., Alba E., Ruíz Y., Cuartero D., Iriarte A., Mora-Luján J.M., Huguet M., Cerdà P., Martínez-Yélamos S. (2021). Catheter-Directed Therapies in Patients with Pulmonary Embolism: Predictive Factors of In-Hospital Mortality and Long-Term Follow-Up. J. Clin. Med..

[37] Visco E., Adamo M., Locantore E., Fiorina C., Chizzola G., Branca L., Abbenante A., Castiello A., Metra M., Curello S. (2019). EkoSonic Endovascular System for Patients with Acute Pulmonary Embolism and Contraindication to Systemic Fibrinolysis. J. Cardiovasc. Med..

[38] Hennemeyer C., Khan A., McGregor H., Moffett C., Woodhead G. (2019). Outcomes of Catheter-Directed Therapy Plus Anticoagulation Versus Anticoagulation Alone for Submassive and Massive Pulmonary Embolism. Am. J. Med..

[39] Tu T., Toma C., Tapson V.F., Adams C., Jaber W.A., Silver M., Khandhar S., Amin R., Weinberg M., Engelhardt T. (2019). A Prospective, Single-Arm, Multicenter Trial of Catheter-Directed Mechanical Thrombectomy for Intermediate-Risk Acute Pulmonary Embolism: The FLARE Study. JACC Cardiovasc. Interv..

[40] Ruzsa Z., Vámosi Z., Berta B., Nemes B., Tóth K., Kovács N., Zima E., Becker D., Merkely B. (2020). Catheter Directed Thrombolytic Therapy and Aspiration Thrombectomy in Intermediate Pulmonary Embolism with Long Term Results. Cardiol. J..

[41] Chauhan M.S., Kawamura A. (2007). Percutaneous Rheolytic Thrombectomy for Large Pulmonary Embolism: A Promising Treatment Option. Catheter. Cardiovasc. Interv..

[42] Arzamendi D., Bilodeau L., Ibrahim R., Noble S., Gallo R., Lavoie-L’allier P., Gosselin G., Deguise P., Ly H., Tanguay J.-F. (2010). Role of Rheolytic Thrombectomy in Massive Pulmonary Embolism with Contraindication to Systemic Thrombolytic Therapy. EuroIntervention.

[43] Semaan D.B., Phillips A.R., Reitz K., Sridharan N., Mulukutla S., Avgerinos E., Eslami M.H., Chaer R. (2023). Improved Long-Term Outcomes with Catheter-Directed Therapies over Medical Management in Patients with Submassive Pulmonary Embolism-a Retrospective Matched Cohort Study. J. Vasc. Surg. Venous Lymphat. Disord..

[44] Yamamoto T., Murai K., Tokita Y., Kato K., Iwasaki Y.-K., Sato N., Tajima H., Mizuno K., Tanaka K. (2009). Thrombolysis with a Novel Modified Tissue-Type Plasminogen Activator, Monteplase, Combined with Catheter-Based Treatment for Major Pulmonary Embolism. Circ. J..

[45] Nakazawa K., Tajima H., Murata S., Kumita S.-I., Yamamoto T., Tanaka K. (2008). Catheter Fragmentation of Acute Massive Pulmonary Thromboembolism: Distal Embolisation and Pulmonary Arterial Pressure Elevation. Br. J. Radiol..

[46] Andresen M., González A., Mercado M., Díaz O., Meneses L., Fava M., Córdova S., Castro R. (2012). Natriuretic Peptide Type-B Can Be a Marker of Reperfusion in Patients with Pulmonary Thromboembolism Subjected to Invasive Treatment. Int. J. Cardiovasc. Imaging.

[47] Meng X., Fu M., Wang J., Xu H. (2022). Effects of Recombinant Human Brain Natriuretic Peptide in Patients with Acute Pulmonary Embolism Complicated with Right Ventricular Dysfunction Who Underwent Catheter-Directed Therapy. Int. Heart J..

[48] Schmitz-Rode T., Janssens U., Schild H.H., Basche S., Hanrath P., Günther R.W. (1998). Fragmentation of Massive Pulmonary Embolism Using a Pigtail Rotation Catheter. Chest.

[49] Akhmerov A., Reich H., Mirocha J., Ramzy D. (2019). Effect of Percutaneous Suction Thromboembolectomy on Improved Right Ventricular Function. Tex. Heart Inst. J..

[50] Sag S., Nas O.F., Kaderli A.A., Ozdemir B., Baran İ., Erdoğan C., Gullulu S., Hakyemez B., Aydinlar A. (2016). Catheter-Directed Ultrasound-Accelerated Thrombolysis May Be Life-Saving in Patients with Massive Pulmonary Embolism after Failed Systemic Thrombolysis. J. Thromb. Thrombolysis.

[51] Zeni P.T., Blank B.G., Peeler D.W. (2003). Use of Rheolytic Thrombectomy in Treatment of Acute Massive Pulmonary Embolism. J. Vasc. Interv. Radiol..

[52] Einarsson F., Sandström C., Svennerholm K., Oras J., Rylander C. (2021). Outcomes of Catheter-Directed Interventions in High-Risk Pulmonary Embolism-a Retrospective Analysis. Acta Anaesthesiol. Scand..

[53] Ferrigno L., Bloch R., Threlkeld J., Demlow T., Kansal R., Karmy-Jones R. (2011). Management of Pulmonary Embolism with Rheolytic Thrombectomy. Can. Respir. J..

[54] Villalba L., Nguyen T., Feitosa R.L., Gunanayagam P., Anning N., Dwight K. (2019). Single-Session Catheter-Directed Lysis Using Adjunctive Power-Pulse Spray with AngioJet for the Treatment of Acute Massive and Submassive Pulmonary Embolism. J. Vasc. Surg..

[55] Lin P.H., Annambhotla S., Bechara C.F., Athamneh H., Weakley S.M., Kobayashi K., Kougias P. (2009). Comparison of Percutaneous Ultrasound-Accelerated Thrombolysis versus Catheter-Directed Thrombolysis in Patients with Acute Massive Pulmonary Embolism. Vascular.

[56] Liang N.L., Chaer R.A., Marone L.K., Singh M.J., Makaroun M.S., Avgerinos E.D. (2017). Midterm Outcomes of Catheter-Directed Interventions for the Treatment of Acute Pulmonary Embolism. Vascular.

[57] Zhou W., Shi H., Yang Z., Liu S., Zhou C., Zhao L., Xia J., Feng Y., Li L. (2009). Value of Percutanous Catheter Fragmentation in the Management of Massive Pulmonary Embolism. Chin. Med. J. (Engl.).

[58] Yoshida M., Inoue I., Kawagoe T., Ishihara M., Shimatani Y., Kurisu S., Kusano K.F., Ohe T. (2006). Novel Percutaneous Catheter Thrombectomy in Acute Massive Pulmonary Embolism: Rotational Bidirectional Thrombectomy (ROBOT). Catheter. Cardiovasc. Interv..

[59] Hubbard J., Saad W.E.A., Sabri S.S., Turba U.C., Angle J.F., Park A.W., Matsumoto A.H. (2011). Rheolytic Thrombectomy with or without Adjunctive Indwelling Pharmacolysis in Patients Presenting with Acute Pulmonary Embolism Presenting with Right Heart Strain and/or Pulseless Electrical Activity. Thrombosis.

[60] Piazza G., Hohlfelder B., Jaff M.R., Ouriel K., Engelhardt T.C., Sterling K.M., Jones N.J., Gurley J.C., Bhatheja R., Kennedy R.J. (2015). A Prospective, Single-Arm, Multicenter Trial of Ultrasound-Facilitated, Catheter-Directed, Low-Dose Fibrinolysis for Acute Massive and Submassive Pulmonary Embolism: The SEATTLE II Study. JACC Cardiovasc. Interv..

[61] Tapson V.F., Sterling K., Jones N., Elder M., Tripathy U., Brower J., Maholic R.L., Ross C.B., Natarajan K., Fong P. (2018). A Randomized Trial of the Optimum Duration of Acoustic Pulse Thrombolysis Procedure in Acute Intermediate-Risk Pulmonary Embolism: The OPTALYSE PE Trial. JACC Cardiovasc. Interv..

[62] Gorgis S., Mawri S., Dabbagh M.F., Aurora L., Ali M., Mitchell G., Jacobsen G., Hegab S., Schwartz S., Kelly B. (2022). Ultrasound-Assisted Catheter-Directed Thrombolysis versus Anticoagulation Alone for Management of Submassive Pulmonary Embolism. J. Cardiol..

[63] Sadeghipour P., Jenab Y., Moosavi J., Hosseini K., Mohebbi B., Hosseinsabet A., Chatterjee S., Pouraliakbar H., Shirani S., Shishehbor M.H. (2022). Catheter-Directed Thrombolysis vs. Anticoagulation in Patients With Acute Intermediate-High-Risk Pulmonary Embolism: The CANARY Randomized Clinical Trial. JAMA Cardiol..

[64] Kroupa J., Buk M., Weichet J., Malikova H., Bartova L., Linkova H., Ionita O., Kozel M., Motovska Z., Kocka V. (2022). A Pilot Randomised Trial of Catheter-Directed Thrombolysis or Standard Anticoagulation for Patients with Intermediate-High Risk Acute Pulmonary Embolism. EuroIntervention.

[65] Kline T.M., Rodino A.M., Dorszynski A., Murray B., Cicci J., Iyer P. (2021). Ultrasound-Assisted Catheter-Directed Thrombolysis versus Systemic Anticoagulation Alone for Submassive Pulmonary Embolism. J. Thromb. Thrombolysis.

[66] D’Auria S., Sezer A., Thoma F., Sharbaugh M., McKibben J., Maholic R., Avgerinos E.D., Rivera-Lebron B.N., Toma C. (2020). Outcomes of Catheter-Directed Thrombolysis vs. Standard Medical Therapy in Patients with Acute Submassive Pulmonary Embolism. Pulm. Circ..

[67] Schissler A.J., Gylnn R.J., Sobieszczyk P.S., Waxman A.B. (2018). Ultrasound-Assisted Catheter-Directed Thrombolysis Compared with Anticoagulation Alone for Treatment of Intermediate-Risk Pulmonary Embolism. Pulm. Circ..

[68] Harrison E., Kim J.S., Lakhter V., Lio K.U., Alashram R., Zhao H., Gupta R., Patel M., Harrison J., Panaro J. (2021). Safety and Efficacy of Catheter Directed Thrombolysis (CDT) in Elderly with Pulmonary Embolism (PE). BMJ Open Respir. Res..

[69] Avgerinos E.D., Liang N.L., El-Shazly O.M., Toma C., Singh M.J., Makaroun M.S., Chaer R.A. (2016). Improved Early Right Ventricular Function Recovery but Increased Complications with Catheter-Directed Interventions Compared with Anticoagulation Alone for Submassive Pulmonary Embolism. J. Vasc. Surg. Venous Lymphat. Disord..

[70] Al-Hakim R., Bhatt A., Benenati J.F. (2017). Continuous Aspiration Mechanical Thrombectomy for the Management of Submassive Pulmonary Embolism: A Single-Center Experience. J. Vasc. Interv. Radiol..

[71] De Gregorio M.A., Guirola J.A., Kuo W.T., Serrano C., Urbano J., Figueredo A.L., Sierre S., Quezada C.A., Barbero E., Jiménez D. (2019). Catheter-Directed Aspiration Thrombectomy and Low-Dose Thrombolysis for Patients with Acute Unstable Pulmonary Embolism: Prospective Outcomes from a PE Registry. Int. J. Cardiol..

[72] Avgerinos E.D., Abou Ali A.N., Liang N.L., Rivera-Lebron B., Toma C., Maholic R., Makaroun M.S., Chaer R.A. (2018). Catheter-Directed Interventions Compared with Systemic Thrombolysis Achieve Improved Ventricular Function Recovery at a Potentially Lower Complication Rate for Acute Pulmonary Embolism. J. Vasc. Surg. Venous Lymphat. Disord..

[73] McIntyre K.M., Sasahara A.A. (1971). The Hemodynamic Response to Pulmonary Embolism in Patients without Prior Cardiopulmonary Disease. Am. J. Cardiol..

[74] Lankhaar J.-W., Westerhof N., Faes T.J.C., Marques K.M.J., Marcus J.T., Postmus P.E., Vonk-Noordegraaf A. (2006). Quantification of Right Ventricular Afterload in Patients with and without Pulmonary Hypertension. Am. J. Physiol. Heart Circ. Physiol..

[75] Mauritz G.-J., Marcus J.T., Westerhof N., Postmus P.E., Vonk-Noordegraaf A. (2011). Prolonged Right Ventricular Post-Systolic Isovolumic Period in Pulmonary Arterial Hypertension Is Not a Reflection of Diastolic Dysfunction. Heart.

[76] Begieneman M.P.V., van de Goot F.R.W., van der Bilt I.A.C., Vonk Noordegraaf A., Spreeuwenberg M.D., Paulus W.J., van Hinsbergh V.W.M., Visser F.C., Niessen H.W.M. (2008). Pulmonary Embolism Causes Endomyocarditis in the Human Heart. Heart.

[77] Toma C., Jaber W.A., Weinberg M.D., Bunte M.C., Khandhar S., Stegman B., Gondi S., Chambers J., Amin R., Leung D.A. (2023). Acute Outcomes for the Full US Cohort of the FLASH Mechanical Thrombectomy Registry in Pulmonary Embolism. EuroIntervention.

[78] Ranade M., Foster M.T., Brady P.S., Sokol S.I., Butty S., Klein A., Maholic R., Safar A., Patel T., Zlotnick D. (2025). Novel Mechanical Aspiration Thrombectomy in Patients With Acute Pulmonary Embolism: Results From the Prospective APEX-AV Trial. J. Soc. Cardiovasc. Angiogr. Interv..

[79] Planer D., Yanko S., Matok I., Paltiel O., Zmiro R., Rotshild V., Amir O., Elbaz-Greener G., Raccah B.H. (2023). Catheter-Directed Thrombolysis Compared with Systemic Thrombolysis and Anticoagulation in Patients with Intermediate- or High-Risk Pulmonary Embolism: Systematic Review and Network Meta-Analysis. CMAJ.

[80] Furfaro D., Stephens R.S., Streiff M.B., Brower R. (2018). Catheter-Directed Thrombolysis for Intermediate-Risk Pulmonary Embolism. Ann. Am. Thorac. Soc..

[81] Giri J., Mahfoud F., Gebauer B., Andersen A., Friedman O., Gandhi R.T., Jaber W.A., Pereira K., West F.M. (2024). PEERLESS II: A Randomized Controlled Trial of Large-Bore Thrombectomy Versus Anticoagulation in Intermediate-Risk Pulmonary Embolism. J. Soc. Cardiovasc. Angiogr. Interv..

[82] Klok F.A., Piazza G., Sharp A.S.P., Ní Ainle F., Jaff M.R., Chauhan N., Patel B., Barco S., Goldhaber S.Z., Kucher N. (2022). Ultrasound-Facilitated, Catheter-Directed Thrombolysis vs. Anticoagulation Alone for Acute Intermediate-High-Risk Pulmonary Embolism: Rationale and Design of the HI-PEITHO Study. Am. Heart J..

[83] Jaber W.A., Gonsalves C.F., Stortecky S., Horr S., Pappas O., Gandhi R.T., Pereira K., Giri J., Khandhar S.J., Ammar K.A. (2025). Large-Bore Mechanical Thrombectomy Versus Catheter-Directed Thrombolysis in the Management of Intermediate-Risk Pulmonary Embolism: Primary Results of the PEERLESS Randomized Controlled Trial. Circulation.

[84] Budaj-Fidecka A., Kurzyna M., Fijałkowska A., Żyłkowska J., Wieteska M., Florczyk M., Szewczyk G., Torbicki A., Filipiak K.J., Opolski G. (2013). In-Hospital Major Bleeding Predicts Mortality in Patients with Pulmonary Embolism: An Analysis of ZATPOL Registry Data. Int. J. Cardiol..

[85] Pietrasik A., Gąsecka A., Szarpak Ł., Pruc M., Kopiec T., Darocha S., Banaszkiewicz M., Niewada M., Grabowski M., Kurzyna M. (2022). Catheter-Based Therapies Decrease Mortality in Patients With Intermediate and High-Risk Pulmonary Embolism: Evidence From Meta-Analysis of 65,589 Patients. Front. Cardiovasc. Med..

[86] Meyer G., Vicaut E., Danays T., Agnelli G., Becattini C., Beyer-Westendorf J., Bluhmki E., Bouvaist H., Brenner B., Couturaud F. (2014). Fibrinolysis for Patients with Intermediate-Risk Pulmonary Embolism. N. Engl. J. Med..

[87] Chatterjee S., Chakraborty A., Weinberg I., Kadakia M., Wilensky R.L., Sardar P., Kumbhani D.J., Mukherjee D., Jaff M.R., Giri J. (2014). Thrombolysis for Pulmonary Embolism and Risk of All-Cause Mortality, Major Bleeding, and Intracranial Hemorrhage: A Meta-Analysis. JAMA.

[88] Monteleone P., Ahern R., Banerjee S., Desai K.R., Kadian-Dodov D., Webber E., Omidvar S., Troy P., Parikh S.A. (2024). Modern Treatment of Pulmonary Embolism (USCDT vs. MT): Results From a Real-World, Big Data Analysis (REAL-PE). J. Soc. Cardiovasc. Angiogr. Interv..

